# Is it necessary to perform a retrosigmoid transposition of the left ureter in Bricker Ileal Conduit surgery?

**DOI:** 10.1186/s12894-022-01073-w

**Published:** 2022-07-27

**Authors:** Jinyou Wang, Zhouting Tuo, Mingzhu Gao, Jie Min, Yi Wang, Tao Zhang, Dexin Yu, Liangkuan Bi

**Affiliations:** 1 Department of Urology, Second Affiliated Hospital of Anhui Medical University, No. 678 Furong Road, Hefei, 200032 People’s Republic of China; 2 Department of Oncology, Second Affiliated Hospital of Anhui Medical University, Hefei, 230601 People’s Republic of China

**Keywords:** Radical cystectomy, Ileal conduit, Ureteroileal anastomotic stricture, Bowel obstruction, Complications

## Abstract

**Background:**

The need for the left ureter to pass through the subsigmoid during ileal conduit diversion surgery has not been investigated in any studies. A modified technique is simply used in the ileal conduit with the left ureter straight over the sigmoid colon due to the possible damage and lack of scientifically validated advantages of this procedure. Our study aimed to investigate the feasibility of the suggested surgical technique, as well as to evaluate perioperative outcomes and postoperative complications with a focus on the prevalence of small bowel obstruction (SBO) and ureteroileal anastomotic stricture (UAS).

**Methods:**

A prospective single-center cohort of 84 consecutive patients undergoing laparoscopic radical cystectomy (LRC) and ileal conduit urinary diversion was conducted between January 2018 and April 2020. The incidence of SBO and UAS, perioperative outcomes, and postoperative complications were compared between a trial group of 30 patients receiving the modified procedure and a control group of 54 patients receiving the conventional Bricker ileal conduit.

**Results:**

The two groups were comparable concerning patient characteristics and clinicopathologic features. No differences were observed in terms of the operation time, perioperative outcomes, and short-term (< 90 days) postoperative complications between the two groups. There were no occurrences of UAS in the modified group, while there were two cases (3.70%) in the patients who received Bricker's ureteroileal anastomosis (*p* = 0.535).

**Conclusion:**

In the present study, a simple and feasible modified technique of ileal conduit is proposed. Compared with traditional techniques, our method has several advantages, including the ability to avoid compression of the left ureter from the mesentery without establishing a retrosigmoid tunnel, a low rate of UAS, and the ability to perform a secondary operation at long-term follow-up.

## Background

Radical cystectomy (RC) and urinary diversion are one of the most challenging procedures in urology. How to simplify the operation, shorten the operation time, and reduce postoperative complications is the direction of urologists’ efforts [1, 2]. The standard Bricker ileal conduit urinary diversion advocates for establishing a retrosigmoid tunnel and contralateral transposition of the left ureter, with the advantage of maintaining the left ureter in an extraperitoneal position and anastomosis with the ileal loop, while potentially reducing disturbance to the bowel [3]. Even for experienced urologists, this step takes at least a quarter-hour to complete. The left ureter needs to be widely freed by 8-12 cm, failing which the ureter's blood supply might be compromised, leading to ischemic stricture of the ureter-ileal anastomosis [4, 5]. The ureter is vulnerable to kinking and extrinsic compression by the mesentery as it travels through the tunnel behind the sigmoid colon. [6–8].

The necessity for the left ureter's passage through the subsigmoid following urine diversion surgery hasn't yet been documented in any studies. Due to the potential harm and the lack of proven benefits of this practice, we implemented a simple modified technique in the ileal conduit and gradually performed ureteroileal anastomosis with the left ureter directly over the sigmoid colon beginning in 2018.

We designed and conducted this non-randomized prospective clinical study with an aim to explore the feasibility of anastomosis of the left ureter directly from the anterior approach of the sigmoid, and also compare perioperative outcomes, complications, and the rate of ureteroileal anastomotic stricture (UAS) in a contemporary series of patients who underwent the modified surgery versus traditional ileal conduit.

## Methods

### Patients

Following institutional review board approval, a single institution prospective chart review was conducted by two experienced urologists on patients who volunteered for RC and ileal conduit surgery from 2018 to 2020. Because our modified intervention was an exploratory technique, we initially planned to enroll only a small population, specifically the study group, and patients with severe obesity (BMI ≥ 35 kg/m^2^), bowel anastomosis, inflammatory bowel disease, distant metastases, renal dysfunction, moderate hydronephrosis, active enteritis, and positive urethral margins were excluded. Billing records were utilized to identify 84 patients who met these criteria. Patients admitted to the hospital in the first month of each quarter were enrolled in the study group and underwent modified surgery and other patients received traditional surgery and were assigned to the control group. Standard neoadjuvant chemotherapy was offered at our institution throughout the study period. The study was conducted in accordance with institutional ethical guidelines, and the informed consent forms were signed by all patients.

### Surgical technique

All operations were performed by the same laparoscopic surgical team (Yu D and Bi L). The laparoscopic radical cystectomy (LRC) and pelvic lymph node dissection were performed by the standard laparoscopic technique [9], and pelvic re-peritonealization was also completed based on the technique previously described [10]. The ileal conduit was constructed according to Bricker’s technique [3], while the left ureter is mobilized and transposed using our ground-breaking method.

**Fig. 1:**
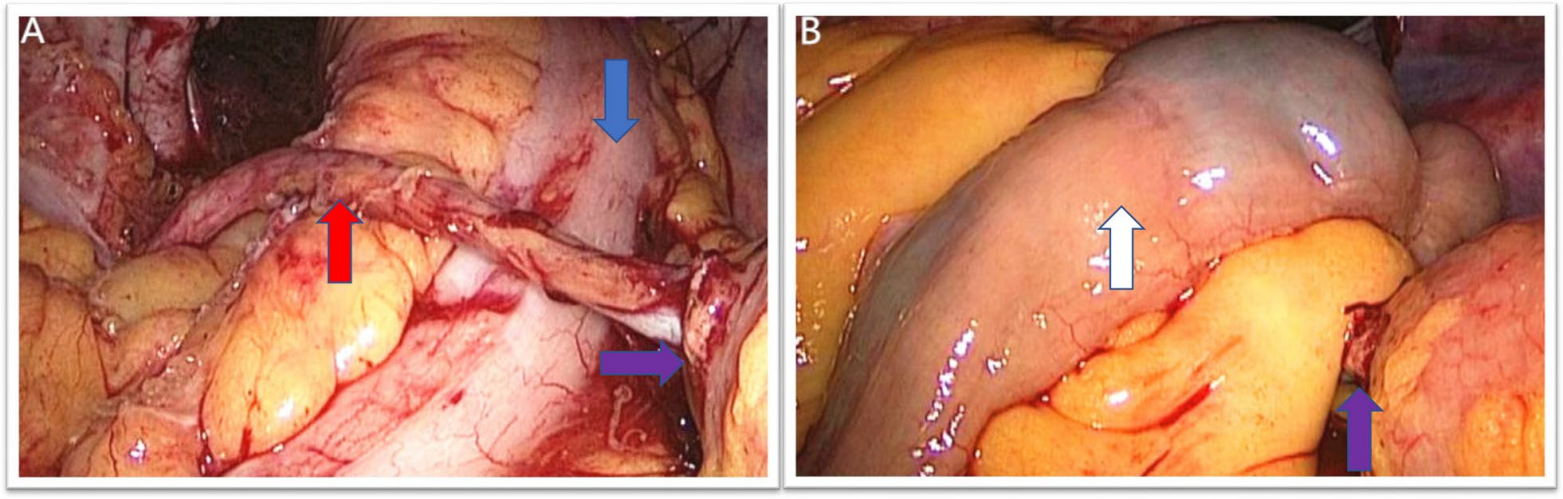
A In our technique, the left ureter (red arrow) passes to the right side over the surface of the sigmoid colon (blue arrow) without establishing a retrosigmoid tunnel, the purple arrow points to the ureteroileal anastomosis. **B** The ileum (white arrow) was finally covered over the left ureter, and the bowel was carefully checked to avoid a visible internal hernia

In the study group, the bilateral ureters were identified and freed from the surrounding tissue as described below. To maximize the blood supply to the ureter, a limited blunt dissection of the ureter was performed, preferably along its lateral aspect, to preserve ureteral adventitia and blood vessels and to avoid any traumatic handling of the distal ureteral tracts. By this approach, the left ureter passed to the right anterior to the sigmoid colon without extensive movement (Fig. 1A). The diversion was performed extracorporeally and a 6-Fr single J-stent was inserted in each of the 2 ureters. Both ureters were implanted using a non-refluxing, split-cuff nipple technique, which included a 0.5 cm longitudinal incision in the distal ureter and turning the ureteral wall over on itself to form a nipple. The ureters were anastomosed to the ileal conduit in an end-to-side fashion with ureteral stents and mounted with 4–0 absorbable Vicryl. Two Single-J stents were placed in the ileal conduit to allow its removal about 2 weeks postoperatively.

In the control group, the left ureter was widely mobilized and transposed to the right side through a retrosigmoid tunnel. The ureteroileal anastomosis was performed according to the Bricker technique [3]. There were no major differences in the surgical procedures between the study and control groups, except for the posterior sigmoid tunnel. All patients received standard postoperative care in our institution.

In both groups, the distal end of the ileal conduit was exteriorized to the skin level, fixed with 1–0 silk sutures, and then the cutaneous stoma was formed in the usual fashion. Finally, the patient was changed to a supine position at 30 degrees with head high and feet low, the ileum was eventually covered over the left ureter, and the bowel was carefully inspected laparoscopically to avoid a visible internal hernia (Fig. 1B). An 18-Ch Foley catheter was placed in the ileal conduit to allow for postoperative flushing and one tubular 24-Ch drain was placed in the cystectomy cavity.

### Postoperative management

All patients received an enhanced recovery after surgery (ERAS) pathway at our institution [11]. The pelvic drain was usually removed on the 7th day postoperatively when no urinary leakage was noted, and the Single-J stents were removed on postoperative day 14. Follow-up was planned according to our institutional protocol and the data were obtained through WeChat, telephone interviews, and outpatient services. Patients were followed up at 1 month postoperatively, every 3 months in the first year, every 6 months in the second year, and annually thereafter. Routine blood, renal function tests, urinary ultrasonography, or computed tomography (CT) were performed at each outpatient follow-up visit.

### Data collection and study outcomes

Data were prospectively collected on demographic and clinical variables, including age, gender, body mass index (BMI), Eastern Cooperative Oncology Group (ECOG) performance status, clinical tumor stage, preoperative upper collecting system status, previous abdominal surgery, smoking status, diabetes mellitus, preoperative mild hydronephrosis, and preoperative creatinine. Moreover, the following intraoperative variables were recorded: operative time, estimated blood loss (EBL), and intraoperative complications. Pathological extension of the primary tumor and lymph node involvement were assigned according to the 2017 TNM staging system [12]. The Society for Fetal Urology (SFU) grading system was used in the rating of hydronephrosis based on the appearance of the renal parenchyma and pelvicalyceal system on CT. Grade 0 is normal; in grade 1, the pelvis was slightly dilated; in grade 2, the calyx was mildly enlarged; in grade 3, the pelvis and calyces were severely dilated, and in grade 4, the parenchyma thickness was reduced and the pelvis and calyces were overly dilated [13].

The primary endpoint of the study was small bowel obstruction (SBO) within 90 days and the occurrence of UAS during long-term follow-up. Complications that occurred within 90 days after surgery was defined as short-term complications or 3 months after surgery was defined as long-term complications. Short-term complications were classified according to the Dindo modification of the Clavien system [14]. UAS was defined as upper collecting system dilatation requiring surgical treatment [6].

### Statistical Analysis

All analyses were performed using SPSS 23.0 (IBM, Armonk, NY, USA) software. Outcomes were expressed as mean and standard deviation or median and/or median and interquartile range (IQR) for continuous variables and as the number of cases and percentage for categorical variables. To compare the differences between the two groups, continuous variables were assessed using the Student t-test or Mann–Whitney U test, and categorical variables were evaluated by the χ2 or fisher exact test. All p values were two-sided, with *p* < 0.05 indicating statistical significance.

## Results

### Patient characteristics

84 patients who underwent LRC and ileal conduit urinary diversion were included in this research. Of them, 54 were treated by the conventional technique, and 30 were treated by the modified technique. Table 1 summarizes baseline patient demographics and preoperative characteristics. We observed a more frequent history of previous abdominal surgery (33.33% vs. 16.67%) in the study group than in the control group, although the difference was not statistically significant (*p *= 0.08). 13 patients had preoperative hydronephrosis (20.00% vs 12.96%, *p* = 0.399). In short, there was no significant difference in baseline characteristics between the two groups.

**Table 1:** Demographic and preoperative characteristics of the 84 patients included in the comparative analysis stratified by transposition of the left ureter in ileal conduit diversion

Variable	Total cases	Study group (n = 30)	Control group (n = 54)	*p* value
Age, [years, mean ± SD]	69.31 ± 9.36	70.84 ± 9.89	68.44 ± 9.03	0.258
Male gender, n (%)	64 (76.20)	26 (86.67)	38 (70.37)	0.158
ECOG performance status, n (%)				0.783
0–1	79 (94.05)	28 (93.33)	51 (94.44)	
> 1	5 (5.95)	2 (6.67)	3 (5.56)	
Diabetes mellitus, n (%)	14 (16.67)	5 (16.67)	9 (16.67)	1.000
BMI, [kg/m2, mean ± SD]	22.77 ± 3.03	22.36 ± 3.04	23.00 ± 3.03	0.359
ASA score, [cases (%)]				0.471
≤ 2	60 (71.43)	20 (66.67)	40 (74.07)	
> 2	24 (28.57)	10 (33.33)	14 (25.93)	
Previous abdominal surgery, n (%)	19 (22.62)	10 (33.33)	9 (16.67)	0.080
Preoperative hydronephrosis, n (%)				0.399
Absent	71 (84.52)	24 (80.00)	47 (87.04)	
Unilateral	7 (8.33)	3 (10.00)	4 (7.41)	
Bilateral	6 (7.14)	3 (10.00)	3 (5.56)	
Clinical tumor stage, n (%)				0.609
≤ T1	28 (33.33)	8 (26.67)	20 (37.04)	
T2	32 (38.10)	13 (43.33)	19 (35.19)	
T3-4	24 (28.57)	9 (30.00)	15 (22.22)	
Preoperative creatinine, [μmol/L, median (range)]	80 (49–143.5)	84 (39–143.5)	78 (44–135)	0.307

ECOG = Eastern Cooperative Oncology Group; BMI = Body mass index; SD = standard deviation

### Operative and pathological outcomes

The operative and pathological outcomes of the two groups are presented in Table 2. 84 individuals underwent LRC and had an ileal conduit with a mean operative time of 271.3 ± 24.03 min in the study group and 279.57 ± 48.47 min in the control group (*p* = 0.299). Patients with postoperative pathological results above T2b were given adjuvant chemotherapy. Operative time, estimated blood loss, pathology type, TNM staging, and postoperative creatinine level were all statistically insignificantly different between the two groups (all *p* > 0.05).

**Table 2:** Operative and pathological characteristics of 84 bladder cancer patients who underwent ileal conduit urinary diversion

Variable	Total cases	Study group (n = 30)	Control group (n = 54)	*p* value
Operative time, [min, mean ± SD]	276.62 ± 41.45	271.3 ± 24.03	279.57 ± 48.47	0.299
EBL, [mL, median (range)]	80 (30–300)	65 (40–300)	90 (30–300)	0.352
Pathologic T stage, [n (%)]				0.166
T1	30 (35.71)	6 (20.00)	24 (44.44)	
T2	32 (38.10)	14 (46.67)	18 (33.33)	
T3	18 (21.43)	8 (26.67)	10 (18.52)	
T4	4 (4.76)	2 (6.67)	2 (3.70)	
Pathological lymph node invasion, n (%)	9 (10.71)	4 (13.33)	5 (9.26)	0.563
Postoperative creatinine, [umol/L, median (range)]	78 (32–179)	81 (38–158)	76 (32–179)	0.501

EBL indicates estimated blood loss; SD = standard deviation

### Ninety-day postoperative complications

Ninety-day postoperative complications were observed in 12 (40.00%) patients who underwent modified technique and in 17 (31.48%) patients who received a traditional surgery (*p* = 0.431; Table 3). Major complications (grade 3–4) were observed in 2 (6.67%) cases in the former group and 3 (5.56%) patients in the latter group (*p* = 0.431). There were 2 and 4 cases of SBO in the study group and control group (*p* = 0.899), respectively, and 1 patient in each of the two groups required reoperation due to severe SBO, with intraoperative ileal and pelvic adhesion and folding, but no enteral hernia. There were 14 (46.67%) cases of hydronephrosis in the study group (3 cases of grade 1, 8 cases of grade 2, 3 cases of grade 3), and 27 (50%) cases in the control group (7 cases of grade 1, 12 cases of grade 2, 8 cases of grade 3) within three months after surgery (*p* = 0.873). At 12 months postoperatively, there was 1 case (3.33%) of grade 1 hydronephrosis in the study group, and 3 (5.56%) cases of hydronephrosis in the control group (1 case of grade 1 and 2 cases of grade 3).

**Table 3:** Overall 90-days postoperative complications in the 84 patients were included in the comparative analysis

Complication type	Total cases	Study group (n = 30)	Control group (n = 54)	*p* value
Bowel obstruction, [n (%)]	6 (7.14)	2 (6.67)	4 (7.41)	0.899
Reoperation, [n (%)]	2 (2.38)	1 (3.33)	1 (1.85)	0.670
Febrile urinary tract infection, [n (%)]	11 (13.10)	5 (16.67)	6 (11.11)	0.470
Wound infection, [n (%)]	5 (5.95)	2 (6.67)	3 (5.56)	0.837
Wound dehiscence, [n (%)]	2 (2.38)	1 (3.33)	1 (1.85)	0.670
Vein thrombosis, [n (%)]	2 (2.38)	1 (3.33)	1 (1.85)	0.670
Sepsis, [n (%)]	2 (2.38)	1 (3.33)	1 (1.85)	0.670
parastomal hernia, [n (%)]	1 (1.19)	0 (0.00)	1 (1.85)	
postoperative hydronephrosis, [n (%)]				0.857
Absent	43 (51.19)	16 (53.33)	27 (50.00)	
Unilateral	15 (17.86)	5 (16.67)	10 (18.52)	
Bilateral	26 (30.95)	9 (30.00)	17 (31.48)	
Hydronephrosis grade, n (%)				0.873
Grade 0	43 (51.19)	16 (53.33)	27 (50.00)	
Grade 1	10 (11.90)	3 (10.00)	7 (12.86)	
Grade 2	20 (23.81)	8 (26.67)	12 (22.22)	
Grade 3	11 (13.10)	3 (10.00)	8 (14.81)	
Grade 4	0 (0.00)	0 (0.00)	0 (0.00)	
90-Days mortality	0 (0.00)	0 (0.00)	0 (0.00)	
Clavien-Dindo classification (< 90d), [n (%)]				0.431
I–II	24 (28.57)	10 (33.33)	14 (25.93)	
≥ III	5 (5.95)	2 (6.67)	3 (5.56)	

### Long-term outcomes

No patients were missed during a minimum of 12 months of follow-up. The Median follow-up time was 29 (range, 13–39) months in the study group, and 27.5 (range, 12–37) months in the control group (*p* = 0.433). There were two (6.67%) cases of tumor recurrence in the study group and two (3.70%) cases in the control group (*p* = 0.541). In the study group, no patients with UAS were observed while in the control group, two (3.7%) patients with UAS were diagnosed at 12 and 16 months after surgery (*p* = 0.750), one on the left side and the other bilaterally. Patients were in gradual remission with surgical treatment. All UAS patients were successfully treated with laparoscopic ureterovesical reimplantation.

## Discussion

To the best of our knowledge, this is the first study to examine the need for retrosigmoid transposition of the left ureter during the Bricker ileal conduit surgery. Our findings confirmed the feasibility of the modified left ureter transposition method in patients undergoing LRC and ileal conduit, and that our modification may simplify the procedure and reduce the incidence of left UAS without increasing bowel complications compared to the traditional Bricker procedure.

Radical cystectomy is a high morbidity procedure, with an overall complication rate of 56–68% after open radical cystectomy (ORC) and 13–19% of advanced complications within 90 days according to the Clavien-Dindo classification [1, 15]. Early complications were expected to be reduced with minimally invasive radical cystectomy [16]. In the present study, the overall and high-grade complication rates were 34.5% and 6.0%, respectively, which were lower than the most recent ORC series. Our study also revealed no significant difference between the two groups in terms of overall and moderate short-term complications, indicating that our modification was safe and feasible.

The study's findings revealed that the rate of postoperative hydronephrosis was higher in the control group than in the study group, with statistically insignificant differences. Postoperative hydronephrosis resolved gradually over time in both groups, which could be attributed to ureteric inflammatory edema or anastomosis. It was critical to protect renal function following ileal conduit surgery because urinary tract obstruction was a major cause of renal damage [17]. These patients' renal function was not clearly impaired throughout our follow-up, and only two patients in the control group experienced UAS.

UAS is a common complication following ileal conduit surgery, with reported incidence in ileal conduit patients ranging from 3 to 15%, which was consistent with the data observed in this study's control group. A greater prevalence of involvement on the left side was also confirmed [4–7]. Most strictures develop within two years following surgery, and they typically have no symptoms for the majority of patients [8]. UAS is classified as benign or malignant, with the majority being benign. The exact etiology of benign strictures is unclear, but many risk factors have been related to the development of strictures, including obesity, perioperative chemotherapy, malnutrition, urinary tract infection, smoking, and so forth [18]. In addition to patient- and disease-related risk factors, ischemic damage to the distal ureter during displacement may result in UAS. In general, the exact surgical method preserves the ureteral blood supply and creates a tension-free anastomosis to assist prevent stricture. An interrupted suture technique and the avoidance of postoperative urine leak after RC are said to significantly reduce the incidence of UAS [7, 8].

Although the rate of UAS has been reduced through careful handling and postoperative care, a higher rate of stenosis has been found in the left ureter than in the right ureter [3], which might be related to increased mobilization and tension of the left ureter passing through the retrosigmoid tunnel, jeopardizing the anastomotic blood supply. In addition, kinking and/or extrinsic compression of the segment of the left ureter below the sigmoid colon may also contribute to ureteral obstruction. For these reasons, various advancements aimed at reducing the aforementioned limitations of the traditional Bricker ileal catheter have been reported [4, 6].

Pagano reported a study of ileal conduit urinary diversion using a modified technique that included an anterior ileal conduit and no left ureteral displacement. The ileal conduit was passed to the left in front of the sigmoid colon in 100 patients, was fastened anteriorly to the sigmoid, and had a greater length than the conventional Bricker ileal conduit, so the left ureters were anastomosed to the conduit each on its naive side. The control group consisted of a historical cohort of 100 patients who received standard Bricker catheters. After a median follow-up of about 5 years in both groups, the UAS rate was 5% in the study group and 15% in the control group. A thorough investigation of perioperative outcomes and postoperative complications was not carried out, UAS laterality was not documented, and ureteral anastomotic leakage occurred in the study group at a rate of 5% [19]. Li described their modified retrosigmoid ileal conduit, which primarily involves contralateral ileal conduit transposition through a retrosigmoid tunnel, and did not report any cases of UAS during the short-term follow-up [4]. In a review of stricture complications in patients undergoing modified retrosigmoid ileal conduit surgery, Ficarra and his colleagues discovered that their modification may contribute to a lower risk of UAS in the short term [6]. However, these modified techniques increased the complexity of the surgery and necessitated the harvesting of a longer ileal segment, which could lead to more electrolyte imbalances and make subsequent looposcopy more challenging. An overlong ileal segment may kink, causing intermittent obstruction of the conduit and recurrent urinary tract infection [20].

In this study, we performed a simple modified technique in ileal conduit following LRC. These individuals who had this modified procedure had a decreased risk of UAS at a median follow-up of 28 months, indicating that avoiding the transposition of the left ureter through the retrosigmoid channel can decrease this complication after surgery. This passage significantly raises the possibility of a stricture [19]. As previously mentioned, the left ureter was prone to kinking, angulation, and compression from the mesentery, resulting in consequent obstruction. Additionally, it is quite challenging to re-operate on UAS patients who were caused by this technique, whether by ureteroscopy or re-laparoscopic surgery. This problem is often treated surgically, with the removal of the stenotic distal ureter and reimplantation of the ureter into a different site of the ileal conduit, with high short- and long-term success rates [7]. However, surgical repair is frequently difficult due to dense adhesions from previous major surgeries or fibrosis caused by radiation treatment. When performing re-laparoscopic surgery on UAS patients, surgeons are confronted with a very short freed distal ureteral segment.

A thorough examination of the colon to exclude an intestinal hernia is a crucial step prior to the completion of our procedure. The first requirement for our modification to be accepted is that it does not increase the likelihood of SBO or intestinal hernia following RC. SBO is the most common complication after radical cystectomy, with an incidence of 9.7–25% [15, 16]. The majority of patients can be cured with conservative care. The present study showed 2 (6.67%) and 4 (7.40%) patients with a bowel obstruction in the modified and control group, respectively. Due to the fact that two patients' symptoms persisted after receiving conservative therapy, and the electrolyte imbalance grew worse over time, they both required reoperation. A severe adhesive intestinal obstruction was discovered postoperatively, but no small intestinal hernia. As a result, the modification of the left ureter passes to the right over the sigmoid colon was both safe and reliable, with no increase in the incidence of SBO or intestinal hernia.

Our modified ileal conduit offered a number of additional benefits over the conventional Bricker ileal conduit. First, our modification was simple to operate and can be accomplished in a relatively short time without extensive peritoneum mobilization. Second, a longer left ureteral segment can be freed if a second laparoscopic operation is performed due to UAS. Future head-to-head comparisons with other modified procedures will be required to outline the advantages and disadvantages of each method.

Several limitations of the present study need to be addressed. First, a contemporaneous historical cohort is used as a control group in this non-randomized study, which also has a small sample size. Second, the follow-up time of the present study was relatively short, which might fail to identify all potential complications of the technique, particularly long-term complications. As this was a small sample univariate analysis comparison, no power analysis was performed, and there was no confidence interval to prevent a negative result from being assessed as a true negative. Further studies are required to confirm the clinical relevance of these findings.

## Conclusions

The Bricker ileal conduit procedure was determined to not require passing the left ureter beneath the sigmoid mesentery based on the aforementioned experience. The suggested procedure provides various benefits over conventional methods, including a low rate of UAS and the ability to prevent compression of the left ureter from the mesentery without creating a retrosigmoid tunnel. However, in order to demonstrate the advantages and long-term effects of this modified ileal conduit, a large-sample prospective randomized controlled study is still necessary.

## Data Availability

The datasets used and/or analyzed during the current study are available from the corresponding author on reasonable request.

## Abbreviations

RC: Radical cystectomy
LRC: Laparoscopic radical cystectomy
UAS: Ureteroileal anastomotic stricture
SBO: Small bowel obstruction
ERAS: Enhanced recovery after surgery
ECOG: Eastern Cooperative Oncology Group
BMI: Body mass index
SD: standard deviation
